# Understanding the Application of Mechanical Dyssynchrony in Patients with Heart Failure Considered for CRT

**DOI:** 10.3390/jcdd11020064

**Published:** 2024-02-17

**Authors:** Abhishek Dutta, Rakan Radwan M. Alqabbani, Andreas Hagendorff, Bhupendar Tayal

**Affiliations:** 1 Department of Cardiology, Nazareth Hospital, Philadelphia, PA 19020, USA; docavishek@gmail.com; 2 Department of Internal Medicine, University Hospitals Cleveland Medical Center, Cleveland, OH 44106, USA; ralqabbani12345@gmail.com; 3 Department of Cardiology, Leipzig University Hospital, 04103 Leipzig, Germany; andreas.hagendorff@medizin.uni-leipzig.de; 4 Harrington and Heart and Vascular Center, University Hospitals Cleveland Medical Center, Cleveland, OH 44106, USA

**Keywords:** cardiac resynchronization therapy, LBBB, echocardiography, dyssynchrony, heart failure

## Abstract

Over the past two decades of CRT use, the failure rate has remained around 30–35%, despite several updates in the guidelines based on the understanding from multiple trials. This review article summarizes the role of mechanical dyssynchrony in the selection of heart failure patients for cardiac resynchronization therapy. Understanding the application of mechanical dyssynchrony has also evolved during these past two decades. There is no role of lone mechanical dyssynchrony in the patient selection for CRT. However, mechanical dyssynchrony can complement the electrocardiogram and clinical criteria and improve patient selection by reducing the failure rate. An oversimplified approach to mechanical dyssynchrony assessment, such as just estimating time-to-peak delays between segments, should not be used. Instead, methods that can identify the underlying pathophysiology of HF and are representative of a substrate to CRT should be applied.

## 1. Introduction

Cardiac resynchronization therapy (CRT) is a specialized treatment approach designed to improve outcomes for patients who are experiencing symptomatic heart failure (HF) with reduced ejection fraction (HFrEF) and an increased QRS duration [1,2,3]. In addition to guideline-directed medical therapy, CRT has established itself as a viable therapeutic option over the past two decades with specific recommendations in the guidelines [4,5,6]. This device therapy has changed the world of HF management, which was predominantly led by pharmacological therapies, and consistently demonstrated its efficacy in alleviating symptoms, improving quality-adjusted life-years (QALYs), as well as reducing mortality beyond the guideline-directed HF pharmacological management [7].

The current mainstay for patient selection for CRT is HF symptoms and electrocardiogram (ECG) [4,5]. Over the past two decades, the recommendations for the ideal patient selection for CRT have been revised a few times; however, the failure rate of the device is still nearly one-third [8]. This is very challenging for clinicians. Besides the cost burden for the health care system, these devices come with certain risks to the patients due to their invasive nature, including the risk of lead infections, along with non-infectious complications like LV lead displacement, high pacing threshold, and phrenic nerve stimulation [9,10]. Therefore, it is crucial to explore alternative approaches that can supplement patient selection beyond the currently used criteria. The use of imaging is limited to left ventricle (LV) systolic function assessment through LVEF ≤ 35%. The role of mechanical dyssynchrony has been explored in patient selection, but the results have not been encouraging. In the following segment, we will discuss the use of mechanical dyssynchrony in patients with heart failure considered for CRT.

## 2. Mechanical Dyssynchrony in Heart Failure

Mechanical dyssynchrony can be explained as the difference in the timing of contraction or relaxation between the left ventricle (LV) and right ventricle (RV) or between different myocardial segments of the LV (intraventricular dyssynchrony). There are two main components for synchronous contraction: an intact conduction system and a viable and well-perfused myocardium. The conduction system is mainly led by the fast Purkinje conduction system, which rapidly delivers and activates the myocardium with a very small physiological delay. Conduction abnormalities, particularly LBBB or interventricular conduction delay, result in intraventricular dyssynchrony due to the loss of this rapid activation system through the conduction system. LBBB can be heterogeneous, ranging from a complete proximal conduction block to a distal Left bundle branch conduction defect [11]. Viable myocardial cells and intact perfusion are the other important components that can result in delayed contraction of the affected region, causing dyssynchrony. Patients with heart failure often meet one of the abovementioned conditions, and therefore, echocardiographic dyssynchrony is frequently noted [12]. We will provide a summary of different echocardiographic techniques used to measure dyssynchrony and how our understanding evolved during the past two decades of working with the concept of dyssynchrony. We will summarize the dyssynchrony methods in relation to the landmark clinical trials—the Predictors of response to CRT (PROSPECT) trial and the Echocardiography-guided CRT (EchoCRT) trial—and their impact on our understanding of dyssynchrony [13,14].

## 3. Pre-Prospect Trial

Some of the important echocardiographic techniques used prior to the landmark PROSPECT trail are discussed in this segment. Different echocardiographic techniques like spectral Doppler, M-mode, and tissue Doppler imaging (TDI)-based methods were introduced during this time to quantify dyssynchrony, and some of the most important ones are discussed below.

### 3.1. Interventricular Mechanical Dyssynchrony

Interventricular dyssynchrony is one of the earliest methods of dyssynchrony assessment. It is the delay of onset of contraction of one ventricle compared to the other. Applying the spectral Doppler technique, aortic and pulmonary velocity flow curves are acquired, and the delay between the onset of flow from the beginning of the QRS duration is calculated. The interventricular mechanical delay (IVMD) is defined as the difference between the pre-ejection time of the left ventricle and right ventricle from the QRS duration. A delay ≥ 40 ms has been proposed as a marker of interventricular dyssynchrony. Dyssynchrony through IVMD was associated with improved outcomes in the CARE-HF trial and, thus far, is the only marker demonstrated to be associated with outcomes after CRT in a randomized setting [15].

### 3.2. M-Mode

The initial imaging studies on LV mechanical dyssynchrony focused on the measurement of opposite wall delay. M-mode is one of the simplest methods to measure that in the parasternal long and short axis views. Pitzalis et al., in their study in heart failure patients with LBBB, showed that a septal-to-posterior wall motion delay (SPWMD) ≥ 130 msec was associated with improved event-free survival post-CRT [16]. However, the approach was limited by its inability to distinguish between delay due to prior scar and electromechanical delay. Moreover, a low amplitude of the motion can make it difficult to identify the peaks with confidence.

### 3.3. Tissue Doppler Imaging (TD1)-Based Opposing Wall Delay (OWD) Methods

**Figure 1:**
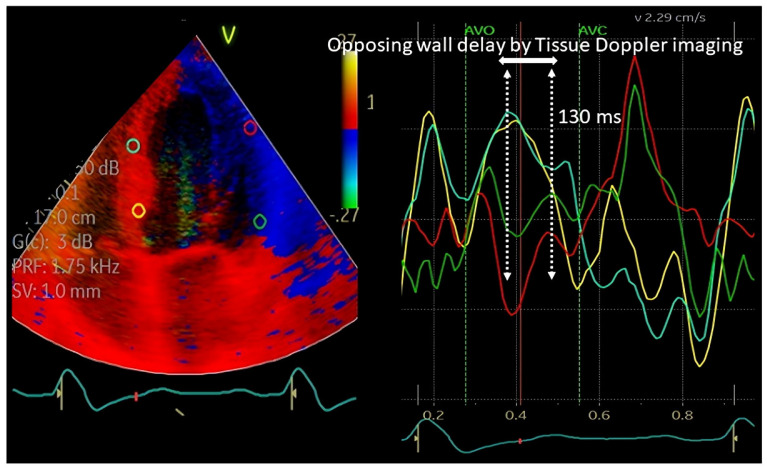
Tissues Doppler Imaging. Regions of interest are placed on the left ventricle in a four-chamber apical view, and delay in the peak velocity of the opposing walls is estimated. Based on the literature, a delay of >65 ms is regarded as a significant delay.

The introduction of the TDI technique provided the ability to measure the time of peak systolic velocity of the LV myocardium at the segmental levels (Figure 1). The advantages of this technique were a good signal-to-noise ratio and the ability to do it offline after the echocardiographic images are acquired. Utilizing this technique, opposing wall delay (OWD) between the septum and the lateral walls can be determined. A cutoff of 65 ms of OWD was shown to be associated with an excellent prognosis after CRT implantation [17]. Another method based on TDI was the assessment of the standard deviation (SD) of the time-to-peak velocity of 12 LV segments in the apical views (excluding the apical segments). A cutoff of >32.6 ms was proposed as an optimal SD found to be associated with improved reverse remodeling after CRT [18].

## 4. PROSPECT Trial

The PROSPECT trial was a multicenter trial performed at 53 centers across the United States, Europe, and Hong Kong [13]. Echocardiographic data were analyzed at three different core laboratories. Patients with symptomatic HF (LVEF ≤ 35%), NYHA class III and IV, and QRS duration ≥ 130 ms prior to CRT implantation were evaluated using echocardiography. Serial echocardiograms were performed post-CRT implantation, and finally, 286 patients fulfilling the inclusion criteria were included. Altogether, 12 different echocardiographic dyssynchrony markers, the most prevalent at that time based on various echocardiographic techniques, were tested in this study. The outcome was LV reverse remodeling. All the spectral Doppler-based methods, including the IVMD method, M-mode–based SPWMD, and TDI-based OWD, were modestly associated with improved reverse remodeling. In contrast, a large interrater variability was noted in most methods of dyssynchrony assessment. Therefore, the authors concluded that the dyssynchrony methods have limited clinical applications beyond the criteria used for the selection of patients for CRT implantation. However, later, the trial was criticized because of several limitations and drawbacks [19].

## 5. Post-PROSPECT Trial

**Figure 2:**
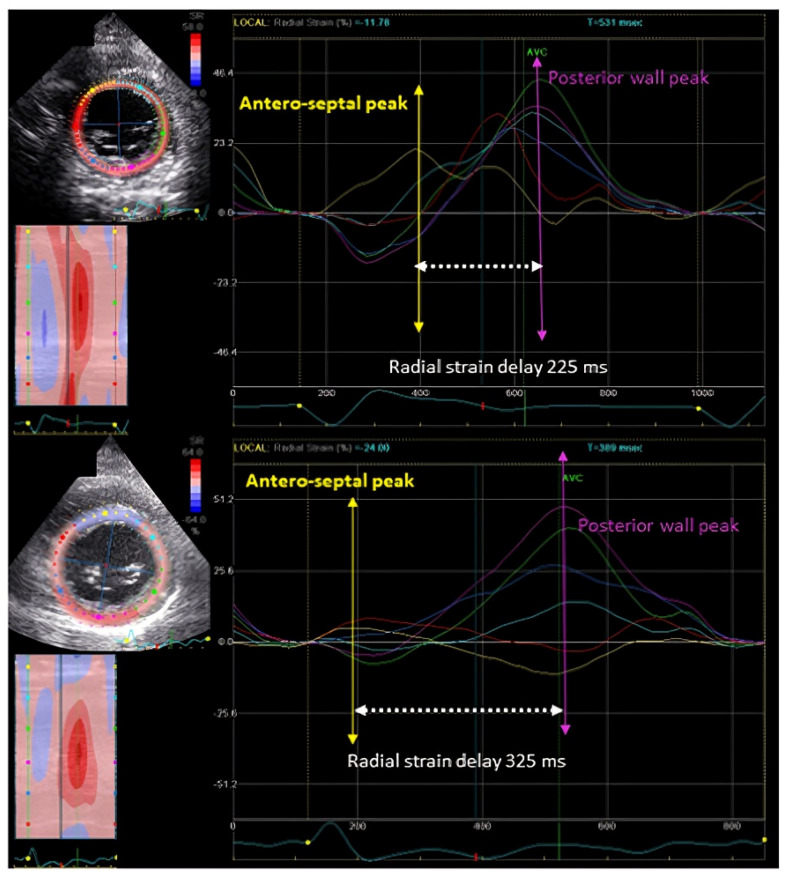
Radial Strain delay. This figure shows two examples of radial strain delay. Both upper and lower panels have a significant delay (≥130 ms) between the antero-septum and the posterior walls of the left ventricle. In addition to significant delay, the lower panel has a typical contraction pattern feature.

After the PROSPECT trial, the main technique applied for dyssynchrony assessment was based on time-to-peak strain assessment through speckle tracking echocardiography (STE). With this technique, segmental myocardial deformation can be assessed rather than the motion. The advantage of STE is that it can overcome some of the shortcomings of the TDI-based technology, like the tethering effect and angle dependency. It was believed that this technique is the key to the dyssynchrony assessment. The most studied and published method during this time was the time-to-peak septal to posterior wall radial strain delay (Figure 2). A cutoff of delay of ≥130 ms at baseline was found to be associated with a good prognosis post-CRT through this method [20,21].

## 6. EchoCRT Trial

The EchoCRT trial was another landmark trial that challenged the use of mechanical dyssynchrony through echocardiography [14]. EchoCRT was a randomized trial where patients with HF and narrow QRS (n = 809) were randomized to CRT vs. no CRT based on the presence of mechanical dyssynchrony in a 1:1 fashion. This trial was stopped prematurely due to poor outcomes noted in patients randomized to CRT. Time-to-peak–based dyssynchrony methods, radial strain septal to posterior wall delay and TDI OWD, were applied to quantify dyssynchrony in this study. This study provided two important insights. Firstly, lone mechanical dyssynchrony in the absence of electrical dyssynchrony does not represent a substrate to CRT. The second and very unanticipated lesson learned was that CRT can be fatal if implanted in the wrong candidate. There was nearly a two-fold increase in the incidence of death, particularly cardiovascular death, among patients randomized to CRT-on. Later, post-hoc studies demonstrated that this might be related to persistent or worsening dyssynchrony post-CRT [22,23].

## 7. Post-EchoCRT Trial: Meaning of Mechanical Dyssynchrony

It was clearly established from the failure of the EchoCRT trial that the presence of electrical dyssynchrony is a must, and most updated guidelines recommended CRT in patients with QRS ≥ 130 ms [4,5]. In fact, Class 1 recommendation is still limited to patients with LBBB morphology with wide QRS ≥ 150 ms, suggesting that only a specific kind of electrical dyssynchrony represents a true substrate for CRT. The role of mechanical dyssynchrony became questionable. We believe that there is certainly a role for mechanical dyssynchrony; however, we need a better understanding of the concept and apply it carefully.

Initial echocardiographic methods largely focused on the time-to-peak–based methods, as explained before. Undoubtedly, the presence of time-to-peak dyssynchrony indicates the presence of mechanical dyssynchrony, but the question arises whether it represents a true substrate for CRT. This can be understood by comparing it to the electrical dyssynchrony estimation through ECG. Widening of QRS suggests electrical dyssynchrony, but not all wide QRS represent a substrate for CRT. Only patients with LBBB represent true electrical substrates for CRT. Similarly, we must focus on identifying dyssynchrony via imaging, which represents a true mechanical substrate. To understand the application of mechanical dyssynchrony, we must understand how LBBB causes HF.

### 7.1. LBBB Cardiomyopathy

Epidemiological studies have demonstrated a significant risk of HF among patients with LBBB, particularly among those with a wide QRS ≥ 150 ms [24,25]. LBBB leads to delayed activation of the LV free wall due to the activation of the LV free wall via myocardial fiber-to-fiber transmission, while the proximal septum is activated early. This abnormal activation leads to a series of changes like inadequate LV filling, increased myocardial work, inadequate LV contraction with reduced septal shortening, and hypoperfusion of the septum, resulting in LV remodeling with dilation and asymmetric LV hypertrophy, eventually leading to HF with reduced LVEF [26]. Patients with LBBB who develop cardiomyopathy due to LBBB theoretically must undergo the abovementioned changes. These patients with LBBB cardiomyopathy typically present with a very typical contraction pattern, where the septum contracts earlier, whereas the LV free wall is stretched, followed by the delayed contraction of the free wall. CRT with an extra lead at the LV free wall synchronizes these abnormal activation patterns, resulting in the reversal of these mechanical delays and improvement of LV function and morphological changes.

**Figure 3:**
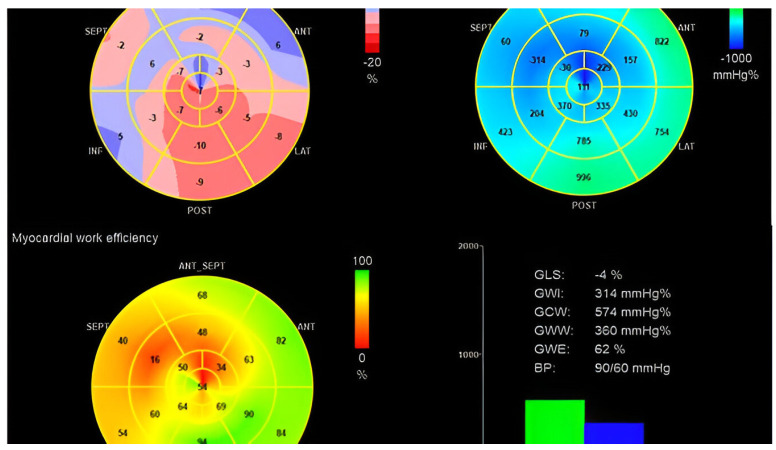
Regional myocardial work in patients with LBBB. This figure shows a patient with LBBB with severely reduced global longitudinal strain (upper left panel). The upper right panel and lower left panel show the regional distribution of myocardial work with severely reduced work index and efficiency in the septum, which is preserved to increase in the LV lateral wall. It is mainly due to the increase in the amount of wasted work performed by the LV (lower right panel).

Recently, the novel approach to estimating the regional LV myocardial work demonstrates how LBBB impacts cardiac mechanics and regional myocardial work with a significant reduction in myocardial work and myocardial efficiency of the septum and an increase in the myocardial work and work efficiency of the lateral wall (Figure 3), which was confirmed using invasive measurements [27]. This study by Russell et al. further demonstrated how CRT normalizes and improves this regional work redistribution by particularly improving the septal work [27]. LV reverse remodeling and CRT response were found to be associated with improvement in global myocardial wasted work and constructive work post-CRT implantation. In fact, the presence of this inefficient regional myocardial performance at baseline was demonstrated to be associated with improved prognosis post-CRT [28]. This inefficient regional myocardial work is due to the ineffective activation of the LV in patients with LBBB, where the septum is contracting earlier prior to the aortic valve opening, and during systole, it is elongating, resulting in wasted work with no contribution to the stroke volume.

LBBB via surface ECG is not perfect, and this is why we need an additional supporting tool to improve patient selection. There are two concerns regarding LBBB via ECG. Firstly, surface ECG may not be accurate and misdiagnose nearly one-third of cases with LBBB. Secondly, LBBB may be a bystander, and HF may be secondary to an ischemic event [29,30]. In these cases, echocardiography can play a role and help isolate cases of ‘true LBBB cardiomyopathy’ where CRT can be beneficial. The role of mechanical dyssynchrony is to tease out the patients with the true electromechanical substrate from patients having LBBB on surface ECG. The authors believe two methods of dyssynchrony assessment, the typical LBBB contraction pattern and apical rocking, can be clinically useful in current practices in isolating the cases of true LBBB cardiomyopathy.

### 7.2. Typical LBBB Contraction Pattern

**Figure 4:**
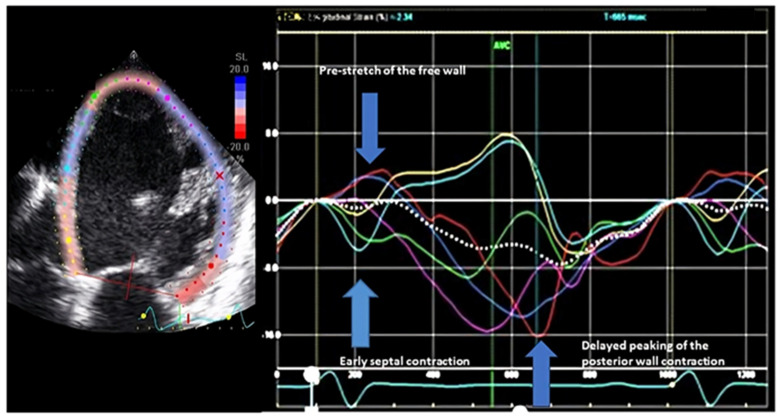
Typical contraction pattern using longitudinal strain. This is an example of a typical left bundle branch contraction pattern showing all three features. It is a simple qualitative technique performed using speckle tracking echocardiography by simply placing regions of interest on the LV in an apical four-chamber image.

The typical LBBB contraction pattern is a qualitative method identified using 2D STE longitudinal strain on the apical four-chamber or long-axis view. It is described to be comprised of three components (Figure 4): (1) early contraction of the septum within 70% of the onset of QRS to the closure of the aortic valve, (2) prestretch of the LV free wall typically before the aortic valve opens, (3) delayed contraction of the LV free wall after the closure of the aortic valve [31,32,33,34]. This is a direct consequence of the abnormal activation of the LV due to LBBB, as described previously. The LV free wall stretch is noted later in the process when the septal contraction becomes very short-lived with advancing cardiomyopathy. Not all patients with LBBB will have these classical features, but when they are present, the response rate with LV reverse remodeling was noted to be 95% [32]. Conversely, the absence of these classical features in patients with LBBB had a 94% failure rate [32]. This study basically establishes that this simple echocardiographic technique can be applied to tease out true LBBB cardiomyopathy, which will respond to CRT. Among those where no contraction pattern is noted, LBBB is possibly a bystander and is not the primary etiology of HF. The importance of this method is further substantiated in a prospective study showing improved survival after CRT implantation in patients with LBBB and a typical LBBB contraction pattern via echo [33]. In this study comprising 208 patients, a three-fold increase in mortality was noted in patients with the absence of a typical LBBB contraction pattern. More importantly, the outcome among patients with relatively narrower QRS duration (130–149 ms) was comparable to those with wider QRS patients (≥150 ms) when this typical LBBB contraction pattern was noted to be present. These relatively narrow complex patients with LBBB currently have Class II recommendations for CRT device implantation; this method can serve as a tool to select these patients for CRT. A recent study comprising the same population demonstrated that the presence of this contraction pattern is associated with a lower risk of complex ventricular arrhythmic events post-CRT [35]. There is strong data supporting the use of this technique. One of the major advantages of this technique is that it is qualitative in nature, and therefore, the reported variability between observers is very low [33]. This was a major critique of the PROSPECT study regarding the dyssynchrony methods. However, most of the data is coming from single-center studies or studies comprising small cohorts. Moreover, most of the published data is from the same group. To further substantiate, larger studies supporting the use of this technique, possibly in randomized settings, are needed. The authors understand that denying CRT to wide QRS and LBBB patients with HF is impossible, but a randomized study in the relatively narrower QRS is possible.

### 7.3. Septal Flash and Apical Rocking

**Figure 5:**
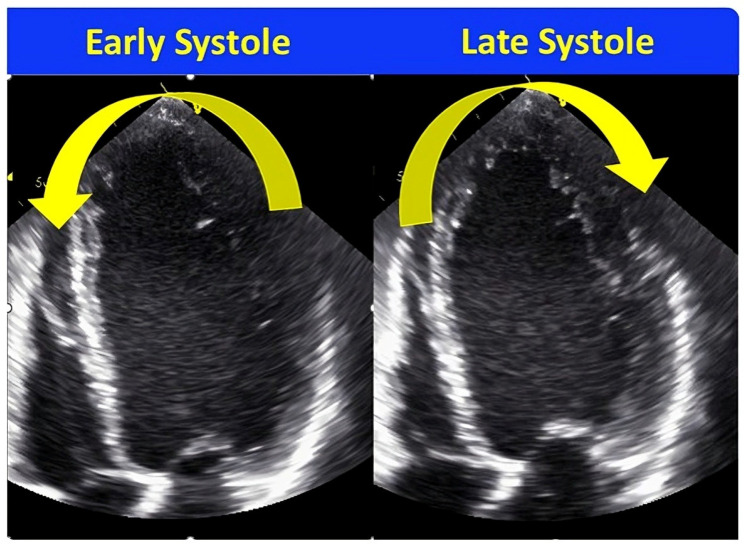
Apical Rocking. Apical rocking is a transverse motion of the LV apex during systole, first towards the septum and later towards the free wall, as shown in the figure. It is a simple method to visually assess the presence of the typical left bundle branch contraction pattern.

This is another qualitative method that has been widely reported in the literature over the past decade [36,37,38,39,40]. Septal flash is a term used to define the brief septal contraction noted in early systole. Apical rocking is the transverse motion of the LV apex during systole, first towards the septum due to early contraction of the septum and then towards the LV free wall due to tugging by the delayed contraction of the LV free wall (Figure 5). It is another simple way of identifying the ineffective contraction pattern noted in patients with LBBB and HF due to abnormal activation. Basically, it is the visual interpretation of the typical LBBB contraction pattern identified using longitudinal strain. The first study was published in 2010, where the authors demonstrated using apical rocking that the accuracy to identify post-CRT responders increased to >80% in comparison to conventional methods with 50–60% accuracy rates. Later, a lot of data was published supporting its use in patients with HF considered for CRT. There are larger multicenter studies showing the application of this method in identifying responders with better accuracy, improved survival, and fewer major adverse cardiac events, as well as improvement in mitral regurgitation post-CRT [36,38,40]. One interesting study comprised 1060 patients who were implanted with CRT. Patients were divided into Class I, Class IIa, and Class IIb based on the guideline recommendations. Mechanical dyssynchrony was defined as the presence of either septal flash or apical rocking, which was assessed prior to device implantation. A higher number of patients with mechanical dyssynchrony demonstrated LV reverse remodeling across all three recommendation groups. The response rate increased from 65% to 77% in the Class I group, 50% to 75% in the Class IIa group, and 38% to 62% in the Class IIb group. The advantage was noted to be much more in patients with Class II recommendation groups and can be of great use in selecting the right patients. There is confusion regarding whether to use septal flash, apical rocking, or both. To clarify, usually, both apical rocking and septal flash are noted simultaneously. In a large series of patients who were implanted with CRT (n = 1058), nearly 60% of patients had either of these two presents in this series; among these, 84% had both, and only 16% had either apical rocking or septal flash [36]. The response rate is also higher when both features are present together. The presence of these signs of septal flash and apical rocking depends on the presence of regional LV scar and LV perfusion; especially, LV lateral wall scar can abolish the presence of the septal flash [41]. This is the reason that many times, these signals may not be present despite having LBBB cardiomyopathy. Although this method is very simple and feasible to assess, proper training is needed as it may be missed by inexperienced eyes. It lacks objectivity, like the demonstration of LV contraction pattern, but both methods identify the same mechanical consequences of abnormal activation patterns in patients with LBBB through different approaches.

## 8. LV Lead Positioning and Latest Site of Activation

Positioning of the LV lead is an important determinant of the outcome after CRT [42,43], but its placement is dependent on the anatomy and distribution of the cardiac veins. Echocardiography can guide in identifying the segments with the site of the latest activation. Using radial strain on the LV short-axis images (base and mid-ventricular level), the timing of each segment’s peak thickening can be calculated from the onset of the QRS duration; in this manner, segments with the latest activation and their adjacent segments can be identified. Two independent randomized trials have shown the benefit of implantation of the LV lead at the site of the latest activation or in the adjacent segments with improved outcomes post-CRT as well as reduced dyssynchrony post-CRT in comparison to those where LV lead is placed remote from the site of latest activation [44,45]. This is an important application of echocardiography; however, it needs to be applied carefully as there can be considerable variability in the assessment of timings for an inexperienced reader.

## 9. AV and VV Delay Optimization

Modern CRT devices allow individualized optimization of the atrioventricular (AV) and ventriculo-ventricular (VV) delays. Although echocardiography plays a central role in these optimizations of AV and VV delays, it is also possible to optimize using device-based intracardiac electrocardiogram [46].

### 9.1. AV Delay

AV delay optimization is primarily used initially for patients with AV block and dual-chamber pacing [47]. The primary issue in patients with CRT is the inter and intra-ventricular conduction delays. However, despite that, AV delay optimization has been utilized in most randomized trials. AV delay optimization can be performed by optimizing LV filling using the mitral inflow pulsed-wave (PW) Doppler filling patterns [48,49] or by optimizing the LV forward flow estimated using the velocity time integral (VTI) of the left ventricular outflow tract [50,51]. There are different approaches for LV filling optimization. The focus of the mitral inflow method is to allow maximum separation of E and A waves without truncation of the A wave. For the LVOT VTI method, the focus is to achieve the maximum VTI for the same heart rate, which suggests increased cardiac output. The importance of AV delay optimization in comparison to a fixed AV delay was tested in a randomized setting in the trial ‘Smart Delay Determined AV Optimization: A Comparison to Other AV Delay Methods Used in Cardiac Resynchronization Therapy (SMART-AV)’ [49]. In this study (n = 980), patients were randomized in a 1:1:1 fashion to three groups: fixed AV delay (120 ms), smart AV delay using an algorithm, and mitral inflow-based echocardiographic method. No change in LV end-systolic volume or HF symptoms was noted at the end of 6 months. Therefore, routine use of AV delay optimization is not recommended. However, in our view, it can be applied to patients who fail to respond to CRT.

### 9.2. VV Delay

The delay in LV contraction relative to the RV contraction is the VV delay. At the very early stage, it was noticed that simultaneous pacing of both ventricles is optimal case in only a few cases (15%), and most patients required some degree of delay individualized for each patient, resulting in an improvement in LV filling, LV output, and mitral regurgitation [52]. Using echocardiographic TDI imaging, sequential optimization of the VV delay was demonstrated to be associated with improved systolic and diastolic function [53]. However, in the randomized setting, only very limited additional benefit was noted from the sequential echocardiographic VV optimization [54].

## 10. Dyssynchrony Post-CRT

Multiple studies have shown that the persistence or worsening of dyssynchrony post-CRT is associated with a poor outcome [22,23,55]. For the post-CRT dyssynchrony assessment, any time-to-peak–based methods can be applied. Worsening or new dyssynchrony could represent either the underlying cardiomyopathy has worsened or the LV lead being placed at the wrong location. Irrespective of the reason, patients should be closely followed, and revision of the lead position or truing of the LV pacing should be considered, although there is no data on whether doing these can help.

## 11. Conclusions

**Figure 6:**
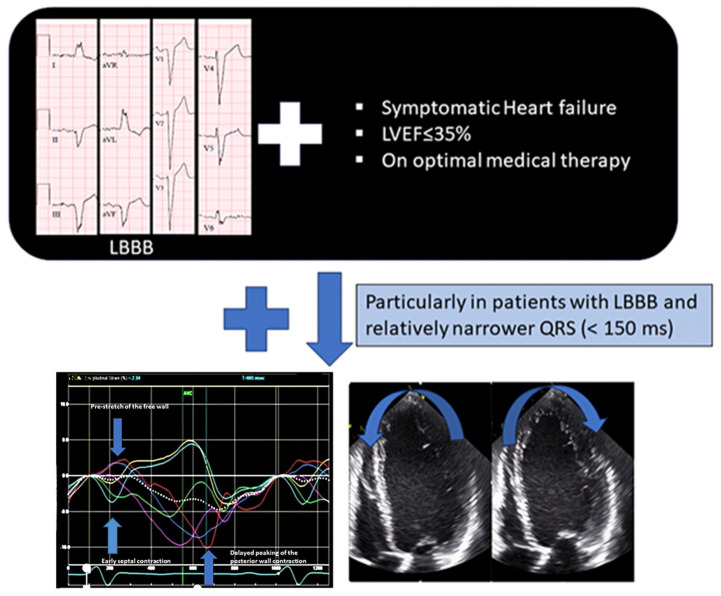
Role of dyssynchrony. Lone mechanical dyssynchrony has no role. However, dyssynchrony can complement the current guidelines, particularly among patients with left bundle branch block and relatively narrower QRS, where it is still a class II recommendation. Simple and robust techniques like the typical left branch block contraction pattern or apical rocking with septal flash can be applied to supplement when in doubt.

Mechanical dyssynchrony via echocardiography has a supportive role in the patient selection for CRT. When patients meet the standard criteria, mechanical dyssynchrony can be utilized to prevent unnecessary device implantation or reduce the failure-to-response rate. It can be particularly applied in patients with relatively narrower QRS complex (Figure 6) and LBBB (<150 ms). Simpler echocardiographic techniques like the typical LBBB contraction pattern and septal flash/apical rocking are more feasible techniques that can be easily performed without complex post-processing analyses. In fact, electrical and mechanical dyssynchrony parameters can be combined to identify optimal patient selection for CRT [56]. Possibly, with advancements in artificial intelligence and machine learning, more robust techniques will be introduced in the future.

## Data Availability

Not applicable.
